# A Multi-Strategy Siberian Tiger Optimization Algorithm for Task Scheduling in Remote Sensing Data Batch Processing

**DOI:** 10.3390/biomimetics9110678

**Published:** 2024-11-06

**Authors:** Ziqi Liu, Yong Xue, Jiaqi Zhao, Wenping Yin, Sheng Zhang, Pei Li, Botao He

**Affiliations:** 1 School of Computer Science and Technology, China University of Mining and Technology, Xuzhou 221116, China; lzq677@cumt.edu.cn (Z.L.); jiaqizhao@cumt.edu.cn (J.Z.); 2 School of Environment and Spatial Informatics, China University of Mining and Technology, Xuzhou 221116, China or wenping.yin@tum.de (W.Y.); shengzhang@cumt.edu.cn (S.Z.); tb22160010a41@cumt.edu.cn (P.L.); mint_tao@cumt.edu.cn (B.H.); 3 Big Geospatial Data Management, Technical University of Munich, 85521 Ottobrunn, Germany

**Keywords:** workflow, task scheduling, remote sensing data, Siberian Tiger Optimization, metaheuristic, optimal assignment

## Abstract

With advancements in integrated space–air–ground global observation capabilities, the volume of remote sensing data is experiencing exponential growth. Traditional computing models can no longer meet the task processing demands brought about by the vast amounts of remote sensing data. As an important means of processing remote sensing data, distributed cluster computing’s task scheduling directly impacts the completion time and the efficiency of computing resource utilization. To enhance task processing efficiency and optimize the allocation of computing resources, this study proposes a Multi-Strategy Improved Siberian Tiger Optimization (MSSTO) algorithm based on the original Siberian Tiger Optimization (STO) algorithm. The MSSTO algorithm integrates the Tent chaotic map, the Lévy flight strategy, Cauchy mutation, and a learning strategy, showing significant advantages in convergence speed and global optimal solution search compared to the STO algorithm. By combining stochastic key encoding schemes and uniform allocation encoding schemes, taking the task scheduling of aerosol optical depth retrieval as a case study, the research results show that the MSSTO algorithm significantly shortens the completion time (21% shorter compared to the original STO algorithm and an average of 15% shorter compared to nine advanced algorithms, such as a particle swarm algorithm and a gray wolf algorithm). It demonstrates superior solution accuracy and convergence speed over various competing algorithms, achieving the optimal execution sequence and machine allocation scheme for task scheduling.

## 1. Introduction

As a crucial means of obtaining information about the Earth’s surface, remote sensing data are widely used in disaster management, smart agriculture, climate and environmental research, forest fire detection, urban management, and many other fields [1,2,3]. Recently, the volume of remote sensing data has surged due to the accumulation of historical data and ongoing improvements in sensor technology, exhibiting distinct “Big Data” characteristics [4,5]. Confronted with the vast quantities of remote sensing data, traditional single-machine computing models can no longer meet the demand for efficient processing, necessitating the adoption of more advanced computing and processing methods. High-performance computing (HPC) refers to the configuration of computing systems and environments with multiple processors or machines, creating clusters or resources connected through different technologies to perform intricate computing tasks more rapidly [5]. As the main computing framework for HPC, distributed cluster computing is extensively employed to handle large-scale remote sensing data [5]. Under the distributed cluster computing framework, workflows can decompose each remote sensing computing task into multiple relatively independent subtasks and allocate these subtasks to different computing nodes for parallel execution, thereby significantly improving computing efficiency [6,7]. However, due to the complexity of the distributed computing environment, workflow task scheduling faces numerous challenges, such as the heterogeneity of computing resources, task dependencies, I/O capabilities, and load balancing. These issues directly impact the execution efficiency of remote sensing tasks and the utilization of resources. Therefore, selecting a scientific scheduling scheme and reasonably utilizing heterogeneous computing resources to maximize computing capabilities has become crucial [8].

In a heterogeneous distributed cluster computing environment, task scheduling for remote sensing data can be considered a variant of the flexible job shop scheduling problem (FJSP) [9]. This problem is fundamentally an intricate NP-hard problem, and traditional mathematical optimization methods struggle to solve it within a reasonable time frame [10,11]. The solution methods for FJSP are mainly divided into heuristic algorithms and metaheuristic algorithms. Heuristic algorithms guide the search direction based on specific rules and can provide relatively good solutions, but they are usually not optimal [12]. Metaheuristic algorithms, as more general heuristic algorithms, are applicable to a wide range of fields and are not tailored to the specific conditions of a problem [9]. Recently, an increasing number of metaheuristic algorithms have been applied to the FJSP problem, including genetic algorithms (GAs) [13,14,15], particle swarm optimization (PSO) [16], ant colony optimization (ACO) [17], and grey wolf optimization (GWO) [18], among others. Many scholars have conducted research on remote sensing task scheduling based on heuristic and metaheuristic algorithms. Zhang et al. [9] introduced a task scheduling approach for managing large volumes of remote sensing data in batch processing to reduce overall completion times. They developed a hierarchical task queue system for optimizing task allocation dynamically, focusing on the workflow granularity in the processing of remote sensing data. Sun et al. [19] proposed a metaheuristic scheduling strategy based on a quantum-inspired evolutionary algorithm (QEA). This method divides tasks based on the data dimensions of remote sensing images and executes scheduling computations within a distributed setting, effectively shortening the total execution time. In addition, Du et al. [20] proposed a workflow task scheduling algorithm that leverages deep reinforcement learning. This algorithm treats the processing of remote sensing data as a scheduling issue within a directed acyclic graph framework, integrating the Markov decision process and methods for calculating fitness. It capitalizes on the strengths of reinforcement learning and deep neural networks to empirically decrease the processing time for remote sensing data.

Despite the achievements of the aforementioned studies, many shortcomings still exist. Heuristic algorithms can obtain effective and relatively good solutions for small-scale problems. However, for large-scale scheduling problems, it is difficult to achieve optimal solutions within an acceptable time frame. In addition, existing metaheuristic algorithms often encounter difficulties escaping local optima and typically exhibit slow convergence rates, which can lead to less-than-ideal performance in task scheduling. The Siberian Tiger Optimization (STO) algorithm is a novel swarm intelligence algorithm that primarily models the hunting and combat tactics of Siberian tigers against bears. It has excellent convergence and optimization capabilities and has been applied in many engineering fields [21]. Viji and Dhanka [22] combined the STO algorithm with an enhanced Wasserstein generative adversarial network to investigate the energy utilization efficiency of hybrid power systems and the lifespan of fuel cells, providing recommendations for optimal control strategies and structural designs. In this study, the STO method was employed to optimize the operational parameters of fuel cell devices. Lakshmiprabha and Kumar [23] proposed a hybrid approach based on the STO algorithm and stacked deep residual networks, analyzing the efficiency and economic evaluation of the integration of pumped storage and alkaline fuel cells, thereby minimizing overall operational costs. Kurapati and Ramachandran [24] introduced a novel convolutional neural accelerator architecture based on the STO algorithm, aimed at enhancing CNN performance in prediction and data broadcasting applications, significantly improving the efficiency of hardware accelerators while reducing power consumption and latency. Additionally, Al-Sarray and Rahebi [25] presented a method that integrates 1D CNN and LSTM networks for detecting attacks in software-defined networks. In the second step of this method, the STO algorithm was applied to enhance the efficiency of the deep learning network, and in the third step, the STO algorithm was utilized for feature selection. This illustrates the widespread application of the STO algorithm across various fields. However, optimizing task scheduling within workflows for batch processing of remote sensing data is a high-dimensional, discrete optimization problem, and the current STO algorithm has certain limitations in addressing such issues. In view of this, this study takes aerosol optical depth retrieval as an example and proposes a Multi-Strategy Improved Siberian Tiger Optimization (MSSTO) algorithm by combining Tent chaotic map, Lévy flight strategy, Cauchy mutation, and learning strategy to improve the original STO algorithm. Subsequently, by introducing the random key encoding scheme (RK) and uniform distribution encoding scheme (UD), the continuous optimization is converted into discrete optimization, effectively addressing the task scheduling problem in remote sensing data batch processing workflows within diverse cluster computing environments. The principal contributions of this research are summarized as follows:

- Proposed the MSSTO algorithm, which integrates Tent chaotic map sequences, Lévy flight strategy, Cauchy mutation, and learning strategy into the STO algorithm, effectively improving the efficiency of finding optimal solutions. Conducted extensive performance comparison analyses, where the MSSTO algorithm was compared with eleven swarm intelligence algorithms, demonstrating its superiority across multiple benchmark problems.
- To make the MSSTO algorithm more suitable for task scheduling in remote sensing data batch processing workflows, this study introduced the RK encoding scheme for task sequences and the UD encoding scheme for machine sequences, achieving an effective mapping from continuous optimization to discrete optimization.
- Optimized the task scheduling process using the MSSTO algorithm, obtaining the optimal sequence of task execution and the best machine allocation scheme, significantly improving the execution efficiency of remote sensing tasks.

The remaining chapters of this paper are organized as follows: Section 2 delineates the problem definition. Section 3 outlines the methodologies. Section 4 assesses the effectiveness of the proposed methods using benchmark test sets. Section 5 introduces the application of remote sensing data task scheduling. Finally, Section 6 provides a summary and outlook of the research.

## 2. Problem Definition

Suppose that the tasks for processing remote sensing data involve *n* images. The processing of each image corresponds to a task unit, with each unit further segmented into *p* sequential processes for completion. In this sequence, each process must wait for the completion of its preceding process before it can start. Within a distributed cluster computing environment, there are *m* computers, denoted as $c_{1},c_{2},c_{2},\ldots ,c_{m}$. The performance differences between each computer primarily stem from variations in their hardware configurations. The makespan of the same process may vary significantly across different hardware configurations. To systematically describe this problem, this paper defines a series of variables and equations to quantify and analyze the execution details of task units on each computer.

(1) $$x\left(t_{ij},t_{ql},c_{k}\right)=\left\{\begin{array}{cc} 1, & \mathrm{if}\;t_{ij}\;\mathrm{is}\;\mathrm{executed}\;\mathrm{before}\;t_{ql}\\ 0, & \mathrm{otherwise} \end{array}\right.$$

where $x(t_{ij},t_{ql},c_{k})$ indicates whether the $j^{th}$ process of task $t_{i}$ is executed before the $l^{th}$ process of task $t_{q}$ on the $k^{th}$ computing node.

(2) $$y\left(t_{ij},c_{k}\right)=\left\{\begin{array}{cc} 1, & \mathrm{if}\;t_{ij}\;\mathrm{is}\;\mathrm{executed}\;\mathrm{by}\;\mathrm{compute}\;\mathrm{node}c_{k}\\ 0, & otherwise \end{array}\right.$$

$y(t_{ij},c_{k})$ represents whether the $j^{th}$ process of task $t_{i}$ is executed on the $k^{th}$ computing node.

The task scheduling in workflows for batch processing of remote sensing data can be viewed as a variant of the FJSP [26]. The primary goal of this scheduling issue is to minimize the makespan, which is to obtain a scheduling strategy that minimizes the completion time of all tasks. This objective can be expressed as follows:

(3) $$makespan=\min(FT_{MAX})$$

Additionally, the scheduling must adhere to the following conditions:

1. The finish time for a process of a task must be at least the total of its start time plus its processing time.
  (4) $$\begin{array}{cc} \; & ST\left(t_{ij}\right)\leq FT\left(t_{ij}\right)+exe\left(t_{ij},c_{k}\right)\times y\left(t_{ij},c_{k}\right),\\ \; & \left(i=1,2,\cdots ,n;j=1,2,\cdots ,p;k=1,2,\cdots ,m\right) \end{array}$$
  where $ST(t_{ij})$ represents the start time of the $j^{th}$ process of task $t_{ij}$, $FT(t_{ij})$ denotes the finish time of the $j^{th}$ process of task $t_{j}$, and $exe(t_{ij},c_{k})$ indicates the execution time of the $j^{th}$ process of task $t_{i}$ on the $k^{th}$ computing node.
2. The start time of a process within the same task must be subsequent to the finish time of the preceding process.
  (5) $$\begin{array}{cc} ST(t_{i(j+1)}) & \geq FT(t_{ij}),\\ (i=1,2,\ldots ,n;\; & j=1,2,\ldots ,p-1) \end{array}$$
3. The finish time of the final process in a task must be earlier than the overall completion time of all tasks.
  (6) $$FT\left(t_{ip}\right)\leq FT_{MAX},\left(i=1,2,\ldots ,n\right)$$
4. At any given time, each computing node is capable of executing only one workflow process.
  (7) $$\begin{array}{cc} ST(t_{ij}) & +exe\left(t_{ij},c_{k}\right)\leq ST\left(t_{ql}\right)+M\left(1-x\left(t_{ij},t_{ql},c_{k}\right)\right)\\ \; & \left(\begin{array}{c} i=1,2,\ldots ,n;j=1,2,\ldots ,p\\ k=1,2,\ldots ,m;q=1,2,\ldots ,n\\ l=1,2,\ldots ,p;M\to \infty \end{array}\right) \end{array}$$
  where $t_{ql}$ represents the $l^{th}$ process of task $t_{q}$.
5. Every workflow process must be carried out by a single computing node exclusively.
  (8) $$\begin{array}{cc} \sum_{k=1}^{m}y\left(t_{ij},c_{k}\right)=1, & \\ (i=1,2,\ldots ,n;\;j=1,2, & \ldots ,p) \end{array}$$

## 3. Methods

### 3.1. Siberian Tiger Optimization

#### 3.1.1. Prey Hunting

In nature, Siberian tigers primarily exhibit two typical behavior patterns: prey hunting and fighting with a bear [21]. In the design of the STO algorithm, the algorithm first simulates the hunting behavior of Siberian tigers for position updating. In this strategy, once a target prey is identified, the Siberian tiger commences an attack and pursues the prey throughout the chase. The prey hunting stage can be segmented into two phase: the attack phase and the chase phase [21].

In the attack phase, the positions of the members in the STO algorithm undergo sudden and extensive changes, thus improving the algorithm’s ability to search globally and explore within the search space. The proposed prey position of each Siberian tiger is determined based on the better-performing members of the population. The proposed prey position is given by the following formula:

(9) $$PP_{i}=\left\{X_{k}|k\in \left\{1,2,\ldots ,N\right\}\wedge F_{k}<F_{i}\right\}\cup \left\{X_{best}\right\}$$

where $PP_{i}$ represents the prey set, $X_{best}$ denotes the current optimal solution, *N* is the total population size, and $F_{i}$ denotes the fitness value for the $i^{th}$ individual. Subsequently, an individual is randomly selected from $PP_{i}$, denoted as $TP_{i}$, to serve as the attack target for the $i^{th}$ member. Finally, the positions of the individual members are updated according to Formula (10).

(10) $$\begin{array}{cc} \; & x_{i,j}^{P1S1}=x_{i,j}+r_{i,j}\cdot \left(TP_{i,j}-I_{i,j}\cdot x_{i,j}\right),\\ & \;\;(i=1,2,\ldots ,N;\;j=1,2,\ldots ,m) \end{array}$$

In which $x_{i,j}^{P1S1}$ indicates the new position of the individual member during the prey hunting attack phase, $x_{i,j}$ represents the value for the $i^{th}$ member in the $j^{th}$ dimension, $r_{i,j}$ is a random value between (0,1), $TP_{i,j}$ indicates the $j^{th}$ dimension of $TP_{i}$, $I_{i,j}$ is a random number selected from the set {1,2}, and *m* represents the dimension of the variables. Subsequently, the new population is obtained according to Formula (11).

(11) $$X_{i}=\left\{\begin{array}{cc} X_{i}^{P1S1} & F_{i}^{P1S1}<F_{i}\\ X_{i} & else \end{array}\right.$$

where $F_{i}^{P1S1}$ denotes the fitness value of $X_{i}^{P1S1}$. In the chase phase, the Siberian tiger enhances the algorithm’s local search and exploitation capabilities by changing its position in the prey attack area, thereby achieving better solutions. This process mainly calculates new positions near the attack location using Formula (12) and obtains the new population using Formula (13).

(12) $$\begin{array}{cc} \; & x_{i,j}^{P1S2}=x_{i,j}+\frac{r_{i,j}\cdot \left(ub-lb\right)}{t},\\ (i=1,2, & \ldots ,N;\;j=1,2,\ldots ,m;\;t=1,2,\ldots ,T) \end{array}$$

where $x_{i,j}^{P1S2}$ indicates the new position of the individual member during the prey hunting chase phase, ub and lb represent the maximum and minimum limits, respectively, and *t* denotes the current iteration number.

(13) $$X_{i}=\left\{\begin{array}{cc} X_{i}^{P1S2} & F_{i}^{P1S2}<F_{i}\\ X_{i} & else \end{array}\right.$$

#### 3.1.2. Fighting with a Bear

Siberian tigers often engage in fights with a bear, primarily to compete for food resources and protect their own safety. Therefore, during the second phase of the STO algorithm, the update strategy of the algorithm’s members simulates the fighting behavior between Siberian tigers and bears.The strategy in this phase is mainly divided into two parts: attack and conflict [21].

In the attack phase, to simulate the Siberian tiger’s attack behavior towards the bear, other members in the algorithm are regarded as potential bears, and a target is randomly selected from them for the attack. This process improves the algorithm’s ability to search globally. The specific formula is as follows:

(14) $$x_{i,j}^{P2S1}=\left\{\begin{array}{cc} x_{i,j}+r_{i,j}\cdot \left(x_{k,j}-I_{i,j}\cdot x_{i,j}\right) & F_{k}<F_{i}\\ x_{i,j}+r_{i,j}\cdot \left(x_{i,j}-I_{i,j}\cdot x_{k,j}\right) & else \end{array}\right.$$

where $x_{k}$ denotes the position of the selected a bear, and *k* is randomly chosen from the set {1,2…i−1,i+1…N}, $x_{i,j}^{P2S1}$ indicates the new position of the individual member in the attack phase of fighting with the bear, and $F_{k}$ represents the fitness value of $x_{k}$. Subsequently, the new population is obtained according to Formula (15).

(15) $$X_{i}=\left\{\begin{array}{cc} X_{i}^{P2S1} & F_{i}^{P2S1}<F_{i}\\ X_{i} & else \end{array}\right.$$

In the conflict phase, the positions of individuals within the STO algorithm experience slight changes, thus boosting the algorithm’s capabilities for local search and exploitation. This process is implemented by Formula (16), and the new population is subsequently obtained using Formula (17).

(16) $$\begin{array}{cc} \; & x_{i,j}^{P2S2}=x_{i,j}+\frac{r_{i,j}\cdot \left(ub-lb\right)}{t},\\ (i=1,2, & \ldots ,N;\;j=1,2,\ldots ,m;\;t=1,2,\ldots ,T) \end{array}$$

where $x_{i,j}^{P2S2}$ indicates the new position of the individual member in the conflict phase of fighting with the bear.

(17) $$X_{i}=\left\{\begin{array}{cc} X_{i}^{P2S2} & F_{i}^{P2S2}<F_{i}\\ X_{i} & else \end{array}\right.$$

### 3.2. Multi-Strategy Improved Siberian Tiger Optimization

Although the STO algorithm has strong optimization capabilities and fast convergence, it can still potentially become trapped in local optima, which restricts its computational precision and could adversely affect the algorithm’s overall performance. To overcome this limitation and enhance the efficiency of the STO algorithm, this study introduces the following four strategies to improve the original algorithm.

#### 3.2.1. Tent Map

In the STO algorithm, candidate solutions are typically initialized using pseudo-random numbers, a strategy that helps optimize the global performance of the algorithm. However, relying solely on pseudo-random numbers for initialization may lead to insufficient exploration of the population, thereby reducing its diversity [27]. To improve the algorithm’s exploration ability and increase population diversity, this study introduces chaotic mapping as a method to improve population initialization. Chaotic mapping transforms the optimization variables into chaotic variables through linear mapping, utilizing their ergodicity and randomness for the optimization search. The final solution is then linearly converted back into the optimization variable space, thereby enhancing the efficiency of the optimization algorithm [28].

Among various chaotic mappings, the Tent map is widely used in many fields due to its excellent uniform ergodic properties [29,30]. As a chaotic mathematical model, the Tent map helps achieve a uniform distribution of the population, thus greatly enhancing the quality of the initial solutions. Its mathematical expression is as follows:

(18) $$Tent_{j+1}=\left\{\begin{array}{cc} \frac{Tent_{j}}{0.59} & 0<Tent_{j}<0.59\\ \frac{1-Tent_{j}}{1-0.59} & 0.59<Tent_{j}<1 \end{array}\right.$$

where $Tent_{j+1}$ represents a value in the interval (0,1). This study introduces the Tent chaotic map in the initial phase of the STO algorithm. The optimized initialization formula is as follows:

(19) $$x_{i,j}=lb+Tent_{i,j}\cdot \left(ub-lb\right)$$

#### 3.2.2. Lévy Flight

In the hunting attack phase of the STO algorithm, a position update is achieved by generating a random step length using a simple uniform random number between the current member and a randomly selected member (representing the bear’s position). Although this method provides direct and uniform exploration within the search space, it may limit the algorithm’s ability to explore extensively, making it difficult for the algorithm to avoid local optima and prone to premature convergence. To enhance global search capabilities and improve exploration efficiency, this study introduces the Lévy flight strategy [31]. The Lévy flight strategy uses the Lévy distribution rather than the conventional uniform or Gaussian distribution to generate step lengths [32]. The Lévy distribution is characterized by a heavy-tailed probability distribution, which allows for occasional long jumps, enabling the algorithm to perform a more extensive search around the current solution [33]. The modified formula after incorporating Lévy flight into Formula (10) is as follows:

(20) $$\begin{array}{cc} \; & x_{i,j}^{P1S1}=x_{i,j}+L_{i,j}\cdot \left(TP_{i,j}-I_{i,j}\cdot x_{i,j}\right),\\ & \;\;(i=1,2,\ldots ,N;\;j=1,2,\ldots ,m) \end{array}$$

where $L_{i,j}$ is generated based on the Lévy distribution using the Mantegna method:

First, generate two independent Gaussian random variables $u∼N(0,\sigma^{2})$ and v∼N(0,1), where σ is given by Formula (21).

(21) $$\sigma ={\left(\frac{\Gamma \left(1+\beta \right)\sin\left(\frac{\pi \beta }{2}\right)}{\Gamma \left(\frac{1+\beta }{2}\right)\beta 2^{\frac{\beta -1}{2}}}\right)}^{\frac{1}{\beta }}$$

where β∈(1,2], typically β=1.5; in this paper, β is also set to 1.5. Next, the Lévy step length $L_{i,j}$ is calculated using the following formula:

(22) $$L_{i,j}=\frac{u}{{\left|v\right|}^{1/\beta }}$$

#### 3.2.3. Cauchy Mutation

In the STO algorithm, traditional local exploration strategies often encounter the dilemma of local optima when dealing with complex optimization problems. To further enhance the algorithm’s exploration capabilities and effectively escape local optima, this paper introduces a probability factor *p* and Cauchy mutation into the local exploration parts of the two main phases of the STO algorithm (the hunting phase and the fighting with brown bears phase). The improved formulas for Formulas (12) and (16) are as follows:

(23) $$\begin{array}{c} x_{i,j}^{S2}=\left\{\begin{array}{cc} X_{best,j}+Cauchy\left(0,1\right)\cdot X_{best,j} & r<p\\ x_{i,j}+\frac{r_{i,j}\cdot \left(ub-lb\right)}{t} & r\geq p \end{array}\right.\\ \;(i=1,2,\ldots ,N;\;j=1,2,\ldots ,m;\;t=1,2,\ldots ,T) \end{array}$$

where $x_{i,j}^{S2}$ indicates the updated position of the individual member, *p* is the introduced probability factor, set to 0.2 in this paper. Additionally, Cauchy(0,1) represents the standard Cauchy distribution function. Cauchy mutation generates random step lengths using the standard Cauchy distribution, making it particularly suitable for scenarios that require wide-range searches. The standard Cauchy distribution is known for its prominent heavy-tailed characteristic, which can generate large random numbers far from the center. This feature is particularly advantageous for exploring under-explored areas of the search space. The formula for the standard Cauchy distribution is as follows:

(24) $$f\left(x\right)=\frac{1}{\pi (1+x^{2})},\;\left(-\infty <x<+\infty \right)$$

#### 3.2.4. Learning Strategy

Within the framework of the original STO algorithm, this paper introduces a novel learning strategy. This strategy aims to promote mutual learning among the Siberian tiger algorithm members after hunting and fighting with brown bears. Specifically, the individual members in the algorithm are divided into four different roles: followers, discoverers, thinkers, and fluctuators [34]. Followers tend to move closer to better-performing individuals; discoverers not only move closer to the best individual but also away from the worst individual; thinkers focus on the differences between the best and worst individuals and strive to narrow this gap; fluctuators exhibit a certain level of volatility, which gradually decreases as the number of iterations increases.Through mutual learning among these different roles, the aim is to enhance the diversity and convergence performance of the STO algorithm, thereby improving its ability to solve high-dimensional optimization problems. The detailed formulas are provided below:

(25) $$x_{i,j}^{P3}=\left\{\begin{array}{cc} x_{i,j}+r_{i,j}\cdot \left(X_{best,j}-x_{i,j}\right) & q\geq \frac{3}{4}\\ x_{i,j}+r_{i,j}\cdot \left(X_{best,j}-x_{i,j}\right)\\ \;-r_{i,j}\cdot \left(X_{worst,j}-x_{i,j}\right) & \frac{1}{2}\leq q<\frac{3}{4}\\ x_{i,j}+r_{i,j}\cdot \left(X_{best,j}-X_{worst,j}\right) & \frac{1}{4}\leq q<\frac{1}{2}\\ x_{i,j}\cdot \left(1+\left(1-\frac{t}{T}\right)\cdot \sin\left(2\pi r\right)\right) & q<\frac{1}{4} \end{array}\right.$$

where $x_{i,j}^{P3}$ represents the updated position of the individual member, $X_{worst,j}$ is the current worst solution, and *T* indicates the total number of iterations. Subsequently, the new population is obtained according to the following formulas:

(26) $$X_{i}=\left\{\begin{array}{cc} X_{i}^{P3} & F_{i}^{P3}<F_{i}\\ X_{i} & else \end{array}\right.$$

The pseudocode for the above model is shown in Algorithm 1.

### 3.3. Continuous to Discrete Encoding Scheme

The MSSTO algorithm is primarily designed for continuous optimization problems; however, task scheduling in remote sensing data batch processing workflows is a discrete problem. To efficiently implement the MSSTO algorithm in the task scheduling for remote sensing data batch processing workflows, this study employs the random key encoding scheme (RK) [35] to achieve an efficient mapping of task sequences from continuous space to discrete space. The mapping of machine sequences is achieved using the uniform distribution encoding scheme (UD).
**Algorithm 1** The Multiple-strategy Siberian Tiger Optimization Algorithm 1: **Input:** The population size pop, maximum number of iterations *T*, problem dimension dim, and objective function. 2: **Output:** The optimal solution achieved by MSSTO. 3: Generate the initialization matrix using Formula (19). 4: Calculate the fitness value for each member of the population. 5: **for** t=1 to *T* **do** 6:    **for** i=1 to pop **do** 7:      Obtain the set of prey for the *i*-th member using Formula (9). 8:      Obtain the Lévy step length $L_{i,j}$ using Formula (22). 9:      Calculate the new position of the $i^{th}$ member in the 1st stage using Formula (20).       $x_{i,j}^{P1S1}\leftarrow x_{i,j}+L_{i,j}\cdot \left(TP_{i,j}-I_{i,j}\cdot x_{i,j}\right)$10:     Update the position of the $i^{th}$ member using Formula (11).11:     Calculate the new position of the $i^{th}$ member in the 2nd stage using Formula (23).
$$x_{i,j}^{P1S2}\leftarrow \left\{\begin{array}{cc} X_{best,j}+Cauchy\left(0,1\right)\cdot X_{best,j} & r<p\\ x_{i,j}+\frac{r_{i,j}\cdot \left(ub-lb\right)}{t} & r\geq p \end{array}\right.$$12:     Update the position of the $i^{th}$ member using Formula (13).13:     Randomly select a member $X_{k}$ as the position of the bear.14:     Calculate the new position of the $i^{th}$ member in the 1st stage using Formula (14).
$$x_{i,j}^{P2S1}\leftarrow \left\{\begin{array}{cc} x_{i,j}+r_{i,j}\cdot \left(x_{k,j}-I_{i,j}\cdot x_{i,j}\right) & F_{k}<F_{i}\\ x_{i,j}+r_{i,j}\cdot \left(x_{i,j}-I_{k,j}\cdot x_{i,j}\right) & else \end{array}\right.$$15:     Update the position of the $i^{th}$ member using Formula (15).16:     Calculate the new position of the $i^{th}$ member in the 2nd stage using Formula (23).
$$x_{i,j}^{P2S2}\leftarrow \left\{\begin{array}{cc} X_{best,j}+Cauchy\left(0,1\right)\cdot X_{best,j} & r<p\\ x_{i,j}+\frac{r_{i,j}\cdot \left(ub-lb\right)}{t} & r\geq p \end{array}\right.$$17:     Update the position of the $i^{th}$ member using Formula (17).18:   **end for**19:   Calculate the new position of the $i^{th}$ member in the 3rd stage using Formula (25).
$$x_{i,j}^{P3}\leftarrow \left\{\begin{array}{cc} x_{i,j}+r_{i,j}\cdot \left(X_{best,j}-x_{i,j}\right) & q\geq \frac{3}{4}\\ x_{i,j}+r_{i,j}\cdot (X_{best,j}-x_{i,j}) & \\ \;\;\;\;-r_{i,j}\cdot \left(X_{worst,j}-x_{i,j}\right) & \frac{1}{2}\leq q<\frac{3}{4}\\ x_{i,j}+r_{i,j}\cdot \left(X_{best,j}-X_{worst,j}\right) & \frac{1}{4}\leq q<\frac{1}{2}\\ x_{i,j}\cdot \left(1+\left(1-\frac{t}{T}\right)\cdot \sin\left(2\pi r\right)\right) & q<\frac{1}{4} \end{array}\right.$$20:   Update the position of the $i^{th}$ member using Formula (26).21:   Save the current best solution $X_{best}$.22:**end for**

**Figure 1:**
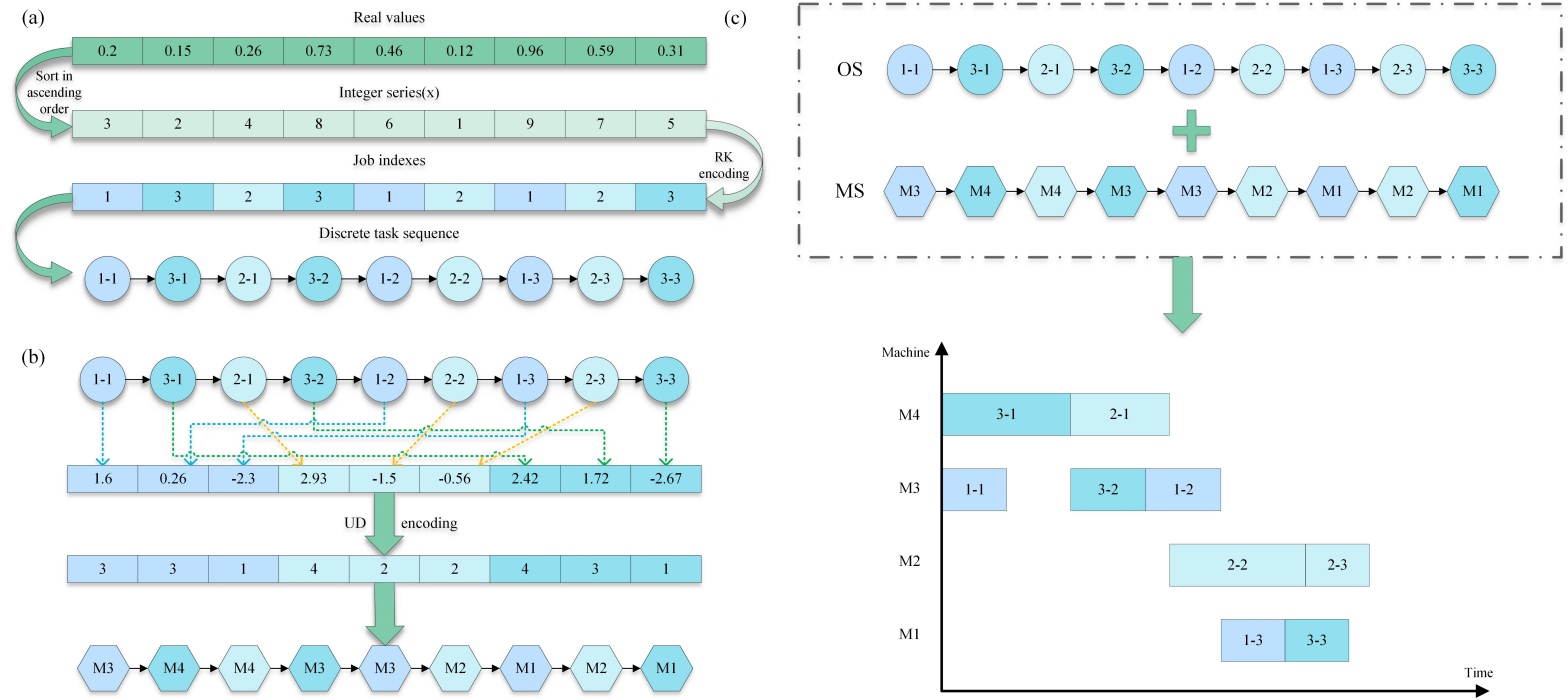
(**a**) RK encoding example diagram; (**b**) machine coding example diagram; (**c**) execute Gantt diagram.

In a task scheduling scenario involving *n* tasks, each containing *p* processes, the dimension length of the MSSTO algorithm is set to 2(n×p). The first (n×p) real numbers are used to represent the task sequence, while the latter (n×p) real numbers are used to represent the machine sequence. The conversion of the task real number sequence to the scheduling operation sequence involves three steps [36]: First, the first (n×p) real numbers obtained by the algorithm are sorted in ascending order. Second, the ranks of these real numbers are displayed as an integer sequence in ascending order. Finally, according to the RK encoding scheme, these ranks are converted into a discrete task sequence. The formula for the RK encoding scheme is as follows:

(27) $$\vec{O}=\left(x_{k}\;\mathrm{mod}\;n\right)+1,\;\left(k=1,2,\ldots ,n\times p\right)$$

where $\vec{O}$ represents the discrete values of the task sequence, $x_{k}$ represents the $k^{th}$ value in the integer sequence, *n* represents the total number of tasks, and *p* indicates the number of processes for each task. For example, suppose there are 3 tasks, each containing 3 processes, i.e., n=3, p=3. For n×p real numbers [0.2,0.15,0.26,0.73,0.46,0.12,0.96,0.59,0.31], as shown in Figure 1a, first sort these real numbers in ascending order. For example, 0.12 is the smallest value and its rank is first, and so on, generating an integer sequence [3,2,4,8,6,1,9,7,5]. Then, the integer sequence is processed according to the RK encoding scheme. Using the first value 3 in the sequence as an example, according to Formula (27), we calculate (3mod3)+1=1. Similarly, the calculation is performed for all integer values to eventually obtain the execution sequence for each process of every task.

After obtaining the execution sequence of the tasks, it is necessary to assign each process of each task to the specified machines for execution. To perform this step, the latter (n×p) real values of the machine sequence need to be mapped to discrete sequence values. This study employs a uniform distribution scheme based on the size of the real values to achieve the mapping from continuous to discrete. The specific steps are as follows: first, the latter (n×p) real values obtained by the algorithm are divided into *n* groups, each containing *p* values. Each group of values corresponds to the processes of a task from the first to the $n^{th}$ task. Second, the machine number corresponding to each real value should be calculated using the following formula:

(28) $$\begin{array}{c} \vec{M}=\left\lfloor \frac{\left(y_{k}+n\right)\cdot \left(m-1\right)}{2n}+1\right\rceil ,\\ \;\left(k=n\times p,n\times p+1,\ldots ,2(n\times p)\right) \end{array}$$

where $\vec{M}$ represents the discrete values of the machine sequence, $y_{k}$ represents the $k^{th}$ value in the real number sequence, $-n\leq y_{k}\leq n$, *m* denotes the total number of machines, *n* indicates the total number of tasks, and *p* refers to the number of processes for each task. Suppose there are currently four machines, M1 to M4, and the execution order of the tasks has been determined according to the RK encoding scheme. Now, using the uniform distribution scheme, map the real values to these four machines. As shown in Figure 1b, there is a set of real numbers [1.6,0.26,−2.3,2.93,−1.5,−0.56,2.42,1.72,−2.67]. First, divide them into three groups based on the number of tasks, with each group containing three processes. For example, the first group is associated with Task 1, the second group with Task 2, and the third group with Task 3. Next, determine on which machine each process should be executed according to Formula (28). For example, for the real number 1.6, using Formula (28), we calculate: $\left\lfloor \frac{\left(1.6+3)\cdot (4-1\right)}{2\times 3}+1\right\rceil =3$. This means that the first process of Task 1 should be executed on machine M3. Using this method, the final machine allocation sequence obtained is [3,3,1,4,2,2,4,3,1]. Finally, by combining the task execution sequence and the machine allocation sequence, a Gantt chart can be generated to show the execution of tasks on machines, as illustrated in Figure 1c.

## 4. Algorithm Performance Evaluation

This section compares the MSSTO algorithm with the STO algorithm and 10 advanced algorithms on the CEC-2017 and CEC-2022 test suites. These 10 advanced algorithms are mainly divided into two categories: (1) highly cited algorithms—particle swarm optimization (PSO) [16], grey wolf optimizer (GWO) [18], whale optimization algorithm (WOA) [37], and African vultures optimization algorithm (AVOA) [38]; (2) advanced algorithms—dung beetle optimizer (DBO) [39], subtraction-average-based optimizer (SABO) [40], golden jackal optimization (GJO) [41], crayfish optimization algorithm (COA) [42], adaptive spiral flying sparrow search algorithm (ASFSSA) [43], and snake optimizer (SO) [44]. The configuration of parameters for the compared algorithms is provided in Table 1. All algorithms have a maximum of 500 iterations, with a population size set to 30. Each algorithm was executed independently 30 times to ensure result stability and reliability. Testing was conducted on a computer equipped with an Intel(R) Core(TM) i7-10700F CPU and 8 GB of RAM, using MATLAB R2018b.

### 4.1. CEC-2017 Evaluation

To thoroughly assess the performance of the MSSTO algorithm, this section employs the CEC-2017 test suite for comparative analysis. The CEC-2017 test suite consists of 29 objective functions. Specifically, C17-F1 to C17-F3 are unimodal functions, C17-F4 to C17-F10 are multimodal functions, C17-F11 to C17-F20 are hybrid functions, and C17-F21 to C17-F30 are composite functions. Note that C17-F2 has been excluded from the test suite due to its instability [45]. When evaluating the algorithms using the CEC-2017 test suite, this study conducted tests at 30, 50, and 100 dimensions.

**Figure 2:**
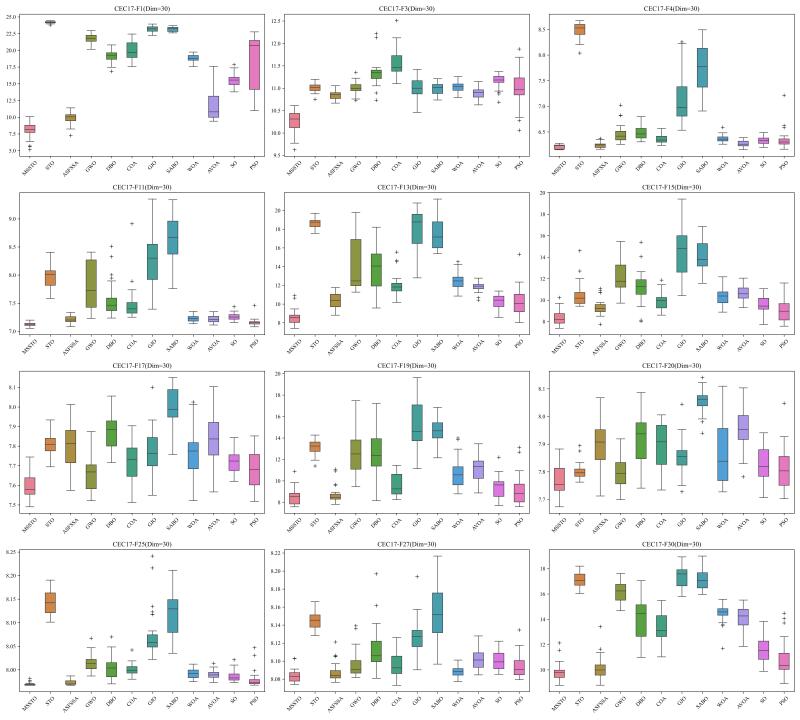
Boxplot of MSSTO and competitor algorithms on the CEC-2017 test suite (dimension = 30).

**Figure 3:**
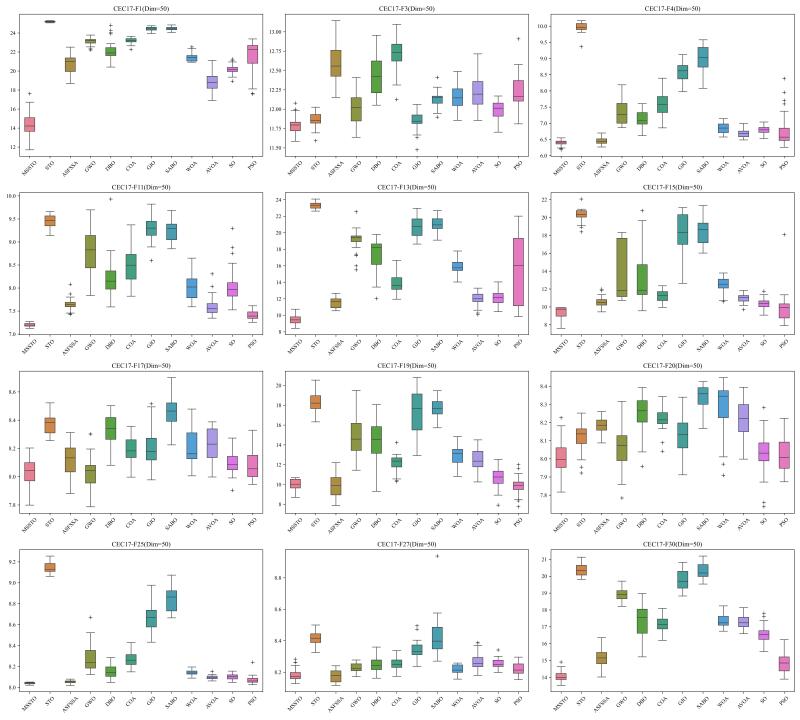
Boxplot of MSSTO and competitor algorithms on the CEC-2017 test suite (dimension = 50).

**Figure 4:**
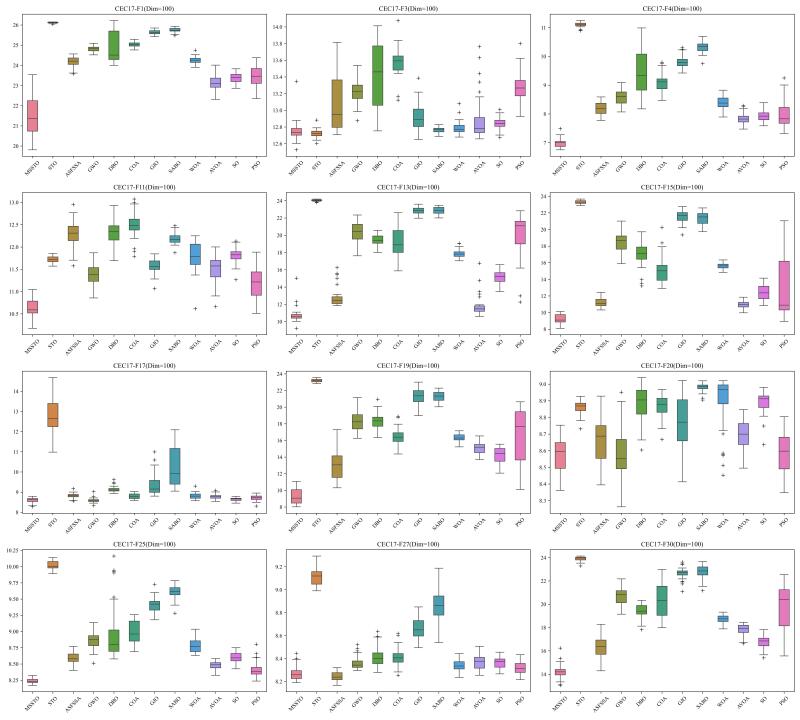
Boxplot of MSSTO and competitor algorithms on the CEC-2017 test suite (dimension = 100).

Figure 2, Figure 3 and Figure 4 present the results of the 12 algorithms on the CEC-2017 test suite at three dimensions in the form of box plots. From these figures, it can be observed that the MSSTO algorithm achieves lower median fitness values on most test functions, indicating its effectiveness in finding better solutions. Additionally, the MSSTO algorithm exhibits smaller variance and fewer outliers, indicating its high consistency and stability across multiple runs. This demonstrates the algorithm’s robustness and adaptability.

**Figure 5:**
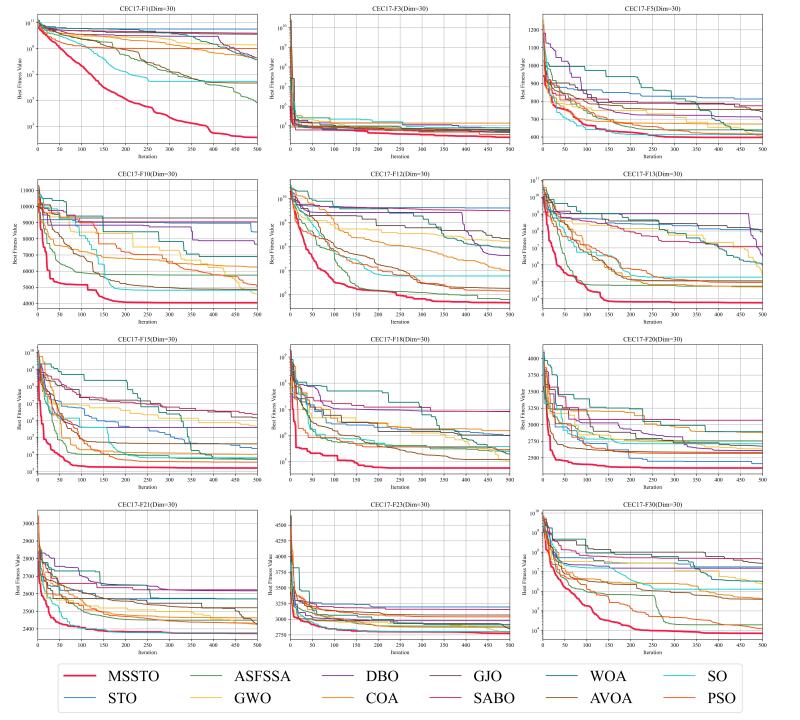
Convergence analysis of the MSSTO and competitor algorithms in CEC-2017 test suite (dimension = 30).

**Figure 6:**
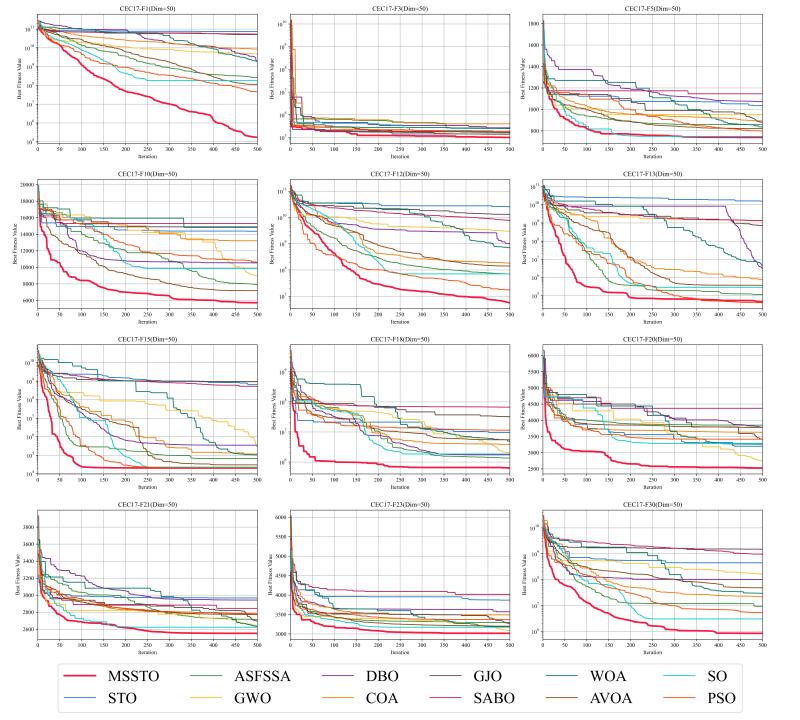
Convergence analysis of the MSSTO and competitor algorithms in CEC-2017 test suite (dimension = 50).

**Figure 7:**
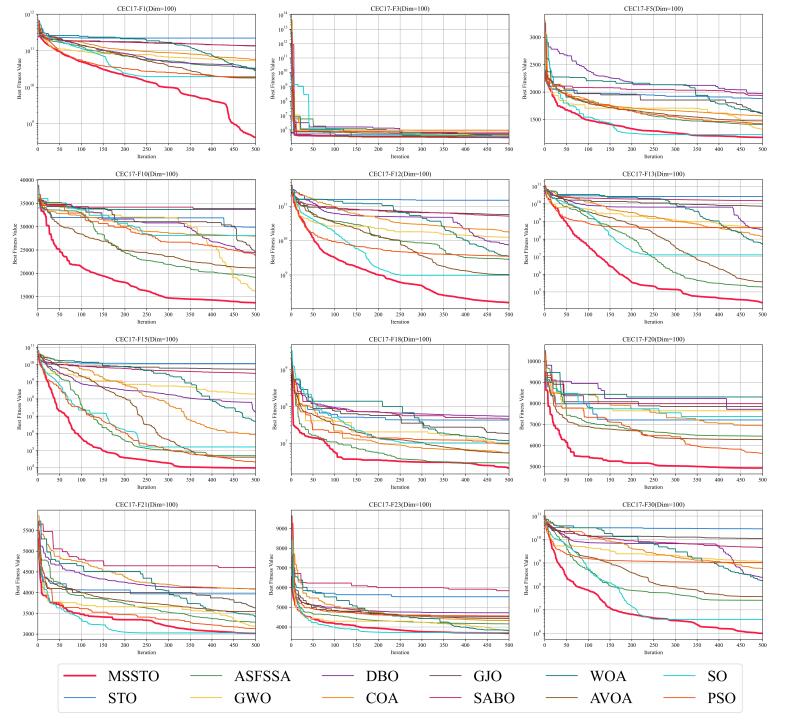
Convergence analysis of the MSSTO and competitor algorithms in CEC-2017 test suite (dimension = 100).

The convergence curves for certain functions from the CEC-2017 test suite are illustrated in Figure 5, Figure 6 and Figure 7. On multiple functions, the MSSTO algorithm converges significantly faster than the other algorithms. For example, on functions F3, F15, and F17, the MSSTO algorithm significantly reduces the fitness value in the initial iteration stages, demonstrating its efficient search capability. Compared to other algorithms, the MSSTO achieves lower final fitness values on most functions, highlighting its advantage in global search capability. Additionally, compared to the STO algorithm, the MSSTO performs better in terms of optimal solution and iteration speed, validating the effectiveness of the various strategies introduced in this study.

### 4.2. CEC-2022 Evaluation

This section further evaluates the MSSTO algorithm using the latest CEC-2022 test suite to highlight its superiority and scalability. The CEC-2022 test suite also consists of unimodal functions (F1), multimodal functions (F2–F5), hybrid functions (F6–F8), and composite functions (F9–F12) [46].

**Figure 8:**
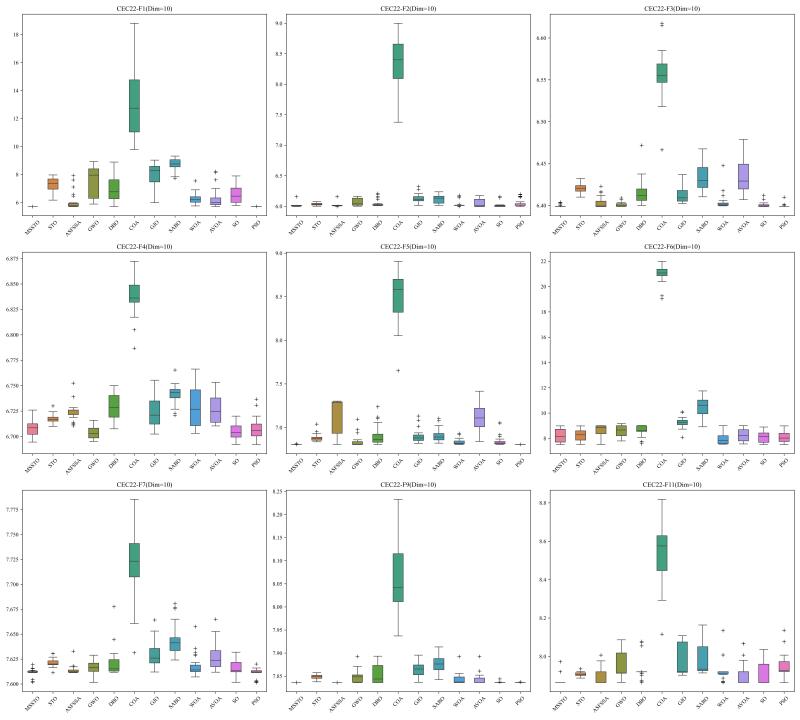
Boxplot of MSSTO and competitor algorithms on the CEC-2022 test suite (dimension = 10).

**Figure 9:**
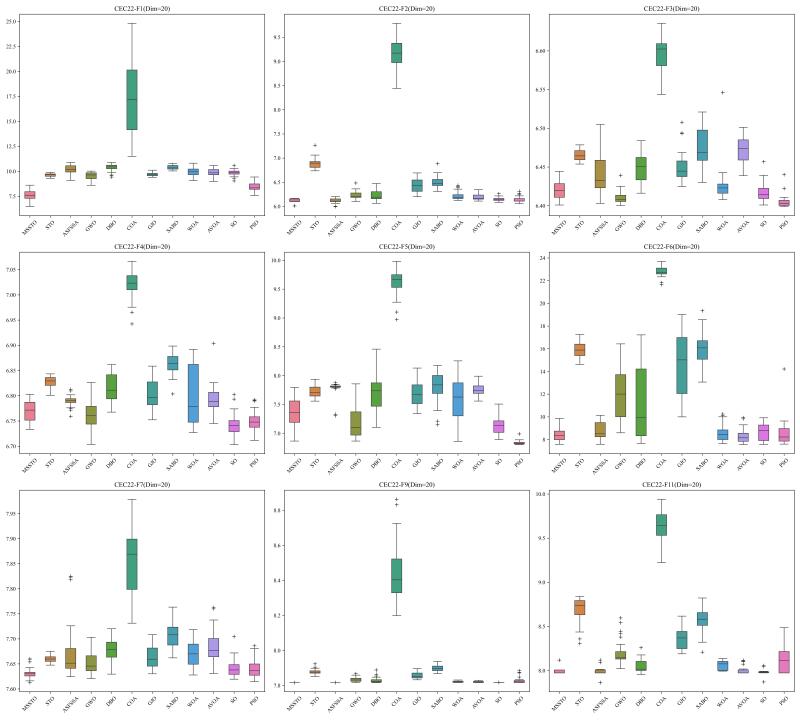
Boxplot of MSSTO and competitor algorithms on the CEC-2022 test suite (dimension = 20).

**Figure 10:**
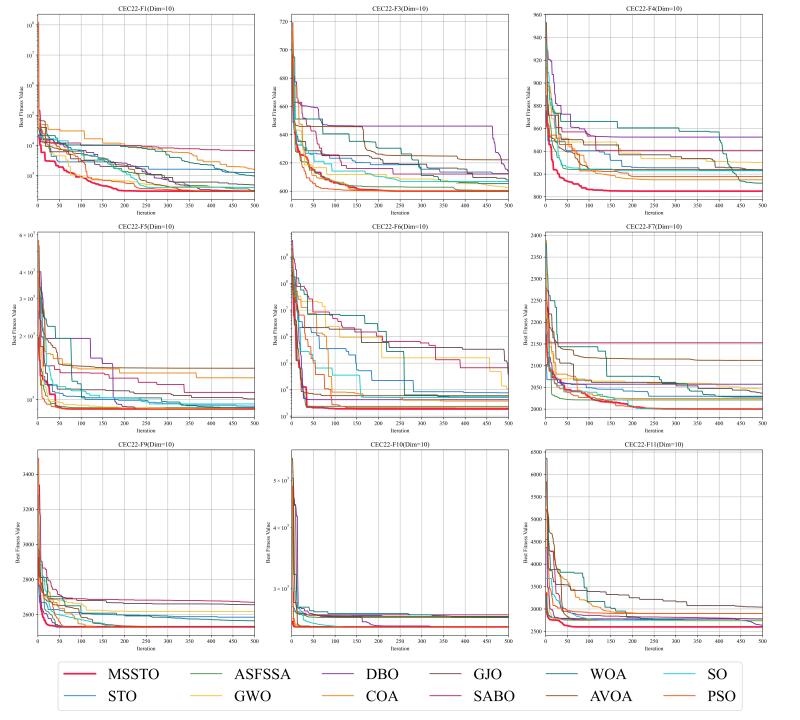
Convergence analysis of the MSSTO and competitor algorithms in CEC-2022 test suite (dimension = 10).

**Figure 11:**
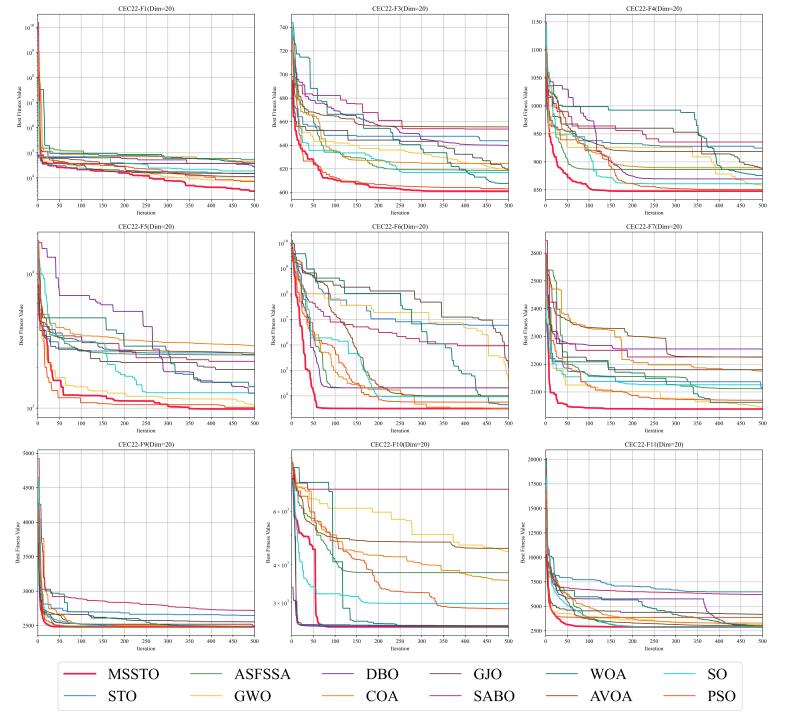
Convergence analysis of the MSSTO and competitor algorithms in CEC-2022 test suite (dimension = 20).

This study tested the MSSTO algorithm in 10-dimensional and 20-dimensional scenarios. Figure 8 and Figure 9 present the box plots for the 10-dimensional and 20-dimensional scenarios, respectively. The figures indicate that the solution quality achieved by the MSSTO algorithm surpasses that of the other competing algorithms on most of the test functions, and the distribution of the solutions demonstrates higher stability. The convergence curves in Figure 10 and Figure 11 reveal that the MSSTO algorithm converges significantly faster than the other compared algorithms on most test functions. This performance suggests that the MSSTO algorithm can swiftly identify superior solutions during the initial phases of the search.

### 4.3. Non-Parametric Test

This section utilizes the Friedman test to perform an in-depth analysis of the experimental outcomes, statistically analyzing the differences between MSSTO and other compared algorithms. The Friedman test can be used to perform statistical ranking on the performance of the MSSTO algorithm and its comparative algorithms on the CEC-2017 and CEC-2022 test suites. The comprehensive results are detailed in Table 2. In the CEC-2017 benchmarks across three dimensions with 29 test functions, the MSSTO algorithm achieved average rankings of 1.97, 1.90, and 1.93, respectively, securing the top overall rank. In the CEC-2022 benchmarks over two dimensions with 12 test functions, the average rankings were 1.58 and 1.92, also leading the overall rankings. These data fully demonstrate the excellent performance and superior capabilities of the MSSTO algorithm in these two test suites.

## 5. Task Scheduling for Remote Sensing Data Batch Processing Workflows

### 5.1. Case Dataset

To confirm the efficacy of the proposed method in remote sensing data batch processing workflow task scheduling, this study selects aerosol optical depth (AOD) retrieval as the experimental case. The experimental data are sourced from MODIS satellite imagery, including MOD02, MYD02, MOD03, MYD03, MOD04_L2, and MYD04_L2. These data are all acquired by sensors on the Aqua and Terra satellites. The geographical coordinates for the output data span from 90° E to 120° E longitude and from 30° N to 50° N latitude, featuring a spatial resolution of 1 km and a temporal resolution of one day.

**Figure 12:**
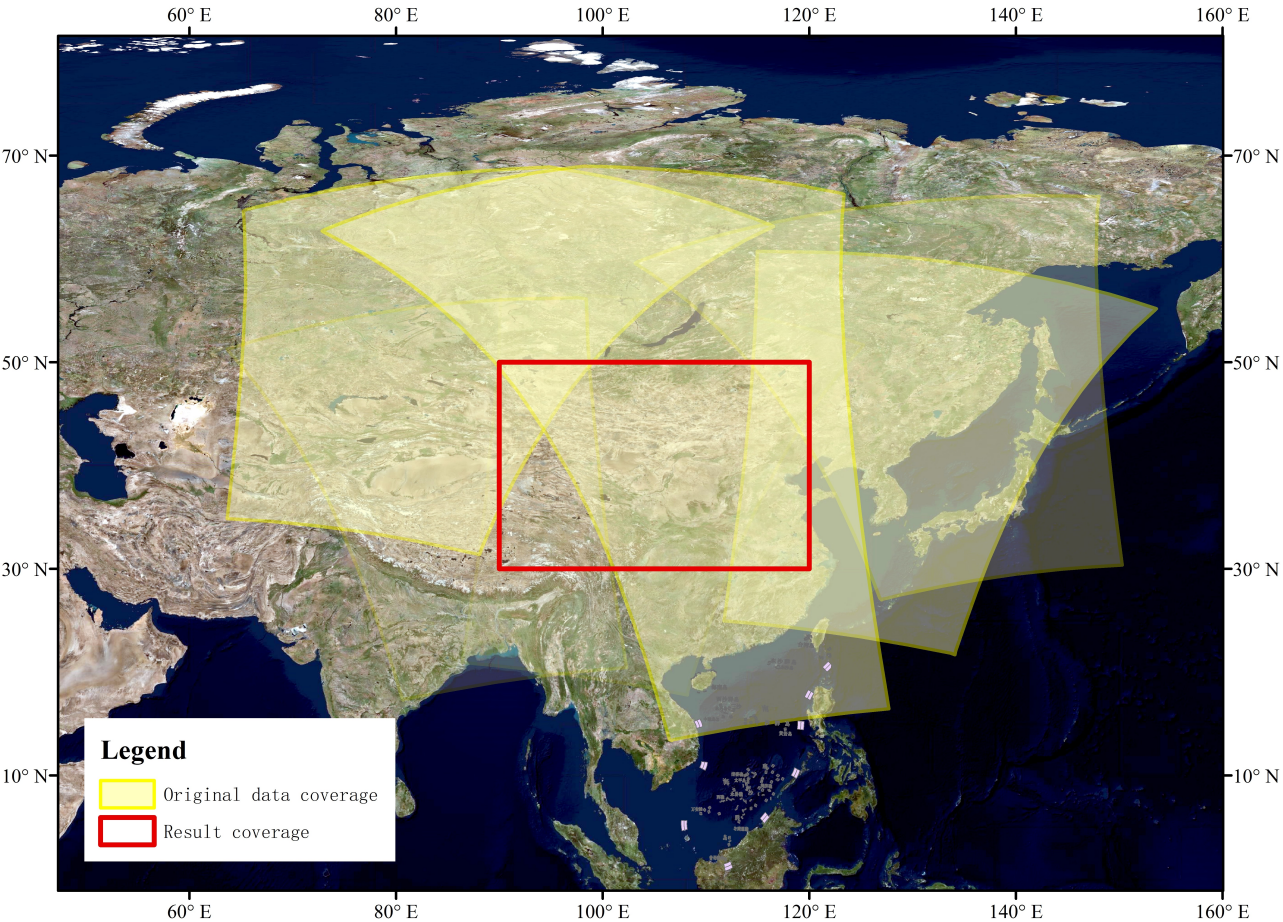
Coverage area of original data and result data.

In this study, the dataset for each day is treated as an independent task unit for processing. To ensure consistency in the experiment, data from the same day are selected to test the time consumption. The AOD retrieval method used in this study is the synergetic retrieval of aerosol properties (SRAP) algorithm [47]. The SRAP algorithm mainly covers key steps such as atmospheric correction, radiometric calibration, cloud masking, geometric correction, image mosaicking, and iterative calculation. The coverage area of the experimental data is shown in Figure 12.

### 5.2. Results and Discussion

According to the SRAP algorithm’s workflow, this study subdivides AOD retrieval into eight processes (p1–p8). The computer hardware configuration used in the experiment is shown in Table 3. The execution time matrix for each workflow on different types of computers is presented in Table 4. To assess the application benefits of the MSSTO algorithm in task scheduling for remote sensing data batch processing workflows, this study contrasts it with the original STO algorithm and nine other swarm intelligence optimization algorithms. These nine algorithms are mainly divided into two categories: (1) highly cited algorithms—PSO [16], GWO [18], WOA [37], and AVOA [38]; (2) advanced algorithms—DBO [39], SABO [40], COA [42], ASFSSA [43], and SO [44]. For all algorithms, the maximum number of iterations is established at 100, with a population size set at 30.

**Figure 13:**
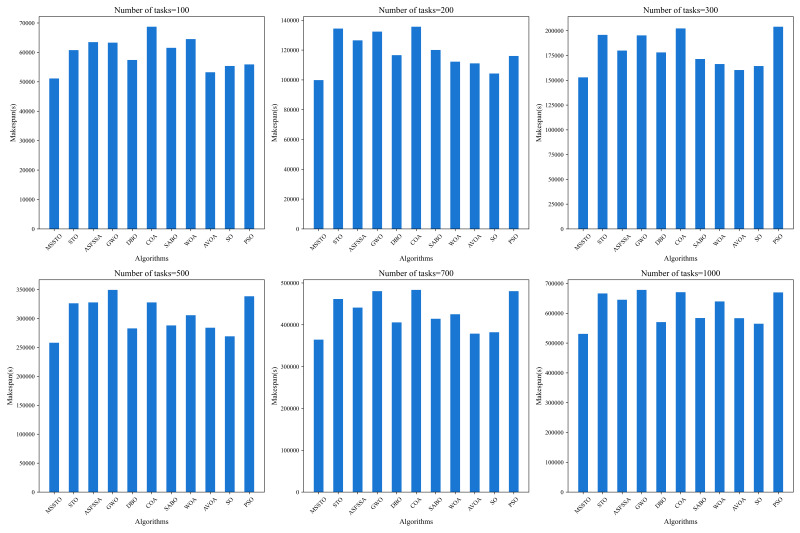
Comparison of makespan with different number of tasks.

Figure 13 presents the statistical results of the completion times for different algorithms when the number of tasks is 100, 200, 300, 500, 700, and 1000, respectively. Clearly, the MSSTO algorithm outperforms all other algorithms in the comparison, exhibiting the shortest completion time. Additionally, Table 5 provides the comparison data of completion times between the MSSTO algorithm and the other compared algorithms. The calculation formula is as follows:

(29) $$Value=\frac{Makespan_{others}-Makespan_{MSSTO}}{Makespan_{MSSTO}}$$

where Value represents the percentage improvement in completion time of the MSSTO algorithm relative to other algorithms, $Makespan_{others}$ indicates the completion time of the compared algorithms, and $Makespan_{MSSTO}$ represents the completion time of the MSSTO algorithm.

**Figure 14:**
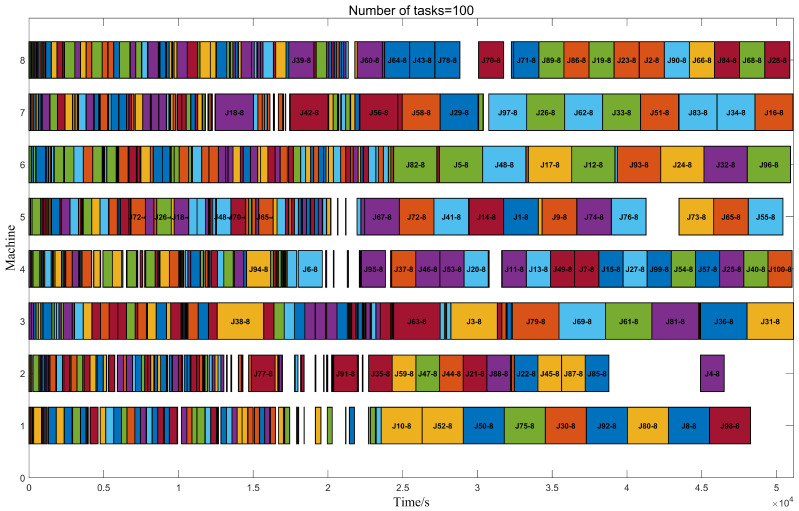
Gantt chart for 100 tasks on eight machines.

From Table 5, when the number of tasks is 100, 200, 300, 500, 700, and 1000, the completion time of the MSSTO algorithm is reduced by 15% to 22% compared to the STO algorithm. Compared to other swarm intelligence algorithms, such as PSO and GWO, the maximum reduction in completion time can reach up to 26%. Even for the better-performing WOA and AVOA algorithms, the completion time shows an improvement of 4% to 10%. These results fully demonstrate that the MSSTO algorithm has significant advantages in reducing task completion time compared to various other algorithms. Figure 14 shows the Gantt chart of the time proportion for processing 100 tasks on eight computers using the MSSTO algorithm combined with the RK task mapping strategy and the UD machine allocation strategy. Through this Gantt chart, it is possible to clearly observe the distribution and time occupancy of different processes of each task on different computers, thereby confirming the significant advantages of the adopted strategies in optimizing task processing time.

## 6. Conclusions

To address the computational pressure brought by the rapid growth of remote sensing big data, optimal scheduling for remote sensing data batch processing workflows is accomplished in a distributed cluster computing environment. This study proposes an improved Multi-Strategy Siberian Tiger Optimization algorithm, which significantly enhances the accuracy and performance of the original STO algorithm by incorporating Tent chaotic mapping, Lévy flight, Cauchy mutation, and learning strategies. Preliminary tests show that the MSSTO algorithm demonstrates higher solution accuracy and a stronger ability to avoid local optima compared to rival algorithms on the CEC-2017 and CEC-2022 benchmark suites.

In the application of task scheduling for remote sensing data batch processing workflows, this study employs the random key encoding scheme and the uniform distribution encoding scheme, combined with the MSSTO algorithm, to achieve the optimal task execution sequence and machine allocation strategy. In this study, we comprehensively considered completion time and I/O performance for task scheduling. Future work should further explore multi-objective integrated scheduling strategies that balance completion time and I/O efficiency. As the task scale increases, the computation time of the MSSTO algorithm also increases. In future work, we will conduct research to reduce the time complexity of the MSSTO algorithm.

Furthermore, the proposed MSSTO algorithm demonstrates significant application potential across various fields, including machine learning, path planning, and image processing. It effectively addresses complex optimization problems, enhancing the execution efficiency and accuracy of various tasks.

## Figures and Tables

**Table 1:** Compare the parameter settings of the algorithms.

Algorithms	Name of the Parameter	Value of the Parameter
PSO	w, c1, c2	0.8, 1.5, 1.5
GWO	a	(0, 2)
WOA	p	0.4
AVOA	L1, L2, w, p1, p2, p3	0.8, 0.2, 2.5, 0.6, 0.4, 0.6
COA	temp	(20, 35)
DBO	P_percent	0.2
GJO	E0, E1	[0, 1], [0, 1.5]
SO	T1, T2, C1, C2, C3	0.25, 0.6, 0.5, 0.05, 2
ASFSSA	P_percent, w	0.2, [0, 1]

**Table 2:** Friedman test results of MSSTO and competitor algorithms on different dimensions of CEC-2017 and CEC-2022.

Suites	CEC-2017							CEC-2022			
**Dim**	**30**		**50**		**100**			**10**		**20**	
**Rank**	**Mean**	**Total**	**Mean**	**Total**	**Mean**	**Total**		**Mean**	**Total**	**Mean**	**Total**
**MSSTO**	**1.97**	**1**	**1.90**	**1**	**1.93**	**1**		**1.58**	**1**	**1.92**	**1**
STO	9.10	9	10.00	11	10.00	11		5.92	7	6.83	8
ASFSSA	4.45	4	4.07	3	4.45	4		4.17	3	5.42	5
GWO	5.48	5	5.00	5	4.86	5		5.83	6	5.00	4
DBO	9.17	11	8.76	10	9.10	10		7.50	9	8.17	10
COA	7.14	8	7.10	8	7.69	8		12.00	12	11.92	12
GJO	9.14	10	8.69	9	8.72	9		8.83	10	7.92	9
SABO	10.69	12	11.10	12	10.55	12		10.75	11	10.75	11
WOA	5.90	6	7.00	7	6.86	7		5.00	5	6.17	7
AVOA	6.86	7	5.83	6	4.41	3		7.42	8	6.00	6
SO	3.28	2	3.45	2	3.69	2		3.67	2	3.00	2
PSO	3.90	3	4.28	4	4.86	5		4.67	4	4.08	3

**Table 3:** Cluster configuration.

Cluster Nodes	Central Processing Unit	Memory	Storage
$c_{1}$	Intel Core i7-12700 (2.10 GHz, 20 CPUs)	32 GB	HDD, SSD
$c_{2}$	Intel Core i5-12400 (2.50 GHz, 12 CPUs)	8 GB	SSD
$c_{3}$	Intel Core i5-11400 (2.60 GHz, 12 CPUs)	16 GB	HDD, SSD
$c_{4}$	Intel Core i5-12400 (2.50 GHz, 12 CPUs)	16 GB	HDD, SSD
$c_{5}$	Intel Core i7-9700 (3.00 GHz, 8 CPUs)	16 GB	HDD, SSD
$c_{6}$	Intel Celeron G5905 (3.50 GHz, 2 CPUs)	8 GB	HDD
$c_{7}$	Intel Core i3-10105 (3.70 GHz, 8 CPUs)	8 GB	SSD
$c_{8}$	Intel Core i3-12100 (3.30 GHz, 8 CPUs)	8 GB	HDD, SSD

**Table 4:** Time overheads of the eight processes of the SRAP algorithm on eight different machines.

	p	Runtime (s)							
**c**		**P1**	**P2**	**P3**	**P4**	**P5**	**P6**	**P7**	**P8**
$c_{1}$		78	526	13	370	28	337	10	2745
$c_{2}$		91	393	10	204	46	185	37	1581
$c_{3}$		156	579	19	688	72	289	70	3110
$c_{4}$		90	424	17	630	74	183	32	1616
$c_{5}$		105	565	26	971	192	259	27	2316
$c_{6}$		151	629	55	484	195	335	164	2895
$c_{7}$		105	501	9	163	38	294	31	2540
$c_{8}$		83	376	28	695	178	183	152	1679

**Table 5:** Makespan optimization percentage of MSSTO compared to competitor algorithms with different numbers of tasks.

MSSTO VS	Number of Tasks					
	**100**	**200**	**300**	**500**	**700**	**1000**
STO	15.8%	25.7%	21.9%	20.9%	21.1%	20.3%
ASFSSA	19.4%	21.1%	15.1%	21.3%	17.4%	17.7%
GWO	19.2%	24.6%	21.7%	26.2%	24.1%	21.8%
DBO	10.9%	14.3%	14.1%	8.8%	10.2%	6.9%
COA	25.6%	26.4%	24.4%	21.3%	24.6%	20.9%
SABO	16.9%	16.8%	10.8%	10.4%	12.0%	9.1%
WOA	20.7%	11.0%	8.1%	15.6%	14.3%	17.0%
AVOA	4.0%	10.1%	4.6%	9.1%	3.8%	9.0%
SO	7.7%	4.2%	7.0%	4.2%	4.6%	6.0%
PSO	8.5%	13.9%	25.1%	23.7%	24.1%	20.8%

## Data Availability

The study did not report any data.
